# Transcriptomic profiling and analysis of differentially expressed genes in asparagus bean (*Vigna unguiculata* ssp. *sesquipedalis*) under salt stress

**DOI:** 10.1371/journal.pone.0219799

**Published:** 2019-07-12

**Authors:** Lei Pan, Xiaolu Yu, Jingjie Shao, Zhichao Liu, Tong Gao, Yu Zheng, Chen Zeng, Chengzhi Liang, Chanyou Chen

**Affiliations:** 1 Hubei Province Engineering Research Center of Legume Plants, School of Life Sciences, Jianghan University, Wuhan, China; 2 Computational Biology Institute and Center for Biomolecular Sciences, Department of Physics, The George Washington University, Washington, DC, United States of America; 3 Institute for Interdisciplinary Research, Jianghan University, Wuhan, China; 4 Institute of Genetics and Development, Chinese Academy of Sciences, Beijing, China; ICAR-Indian Institute of Agricultural Biotechnology, INDIA

## Abstract

Asparagus bean (*Vigna unguiculata* ssp. *sesquipedalis*) is a warm season legume which is widely distributed over subtropical regions and semiarid areas. It is mainly grown as a significant protein source in developing countries. Salinity, as one of the main abiotic stress factors, constrains the normal growth and yield of asparagus bean. This study used two cultivars (a salt-sensitive genotype and a salt-tolerant genotype) under salt stress vs. control to identify salt-stress-induced genes in asparagus bean using RNA sequencing. A total of 692,086,838 high-quality clean reads, assigned to 121,138 unigenes, were obtained from control and salt-treated libraries. Then, 216 root-derived DEGs (differentially expressed genes) and 127 leaf-derived DEGs were identified under salt stress between the two cultivars. Of these DEGs, thirteen were assigned to six transcription factors (TFs), including AP2/EREBP, CCHC(Zn), C2H2, WRKY, WD40-like and LIM. GO analysis indicated four DEGs might take effects on the “oxidation reduction”, “transport” and “signal transduction” process. Moreover, expression of nine randomly-chosen DEGs was verified by quantitative real-time-PCR (qRT-PCR) analysis. Predicted function of the nine tested DEGs was mainly involved in the KEGG pathway of cation transport, response to osmotic stress, and phosphorelay signal transduction system. A salt-stress-related pathway of “SNARE interactions in vesicular transport” was concerned. As byproducts, 15, 321 microsatellite markers were found in all the unigenes, and 17 SNP linked to six salt-stress induced DEGs were revealed. These candidate genes provide novel insights for understanding the salt tolerance mechanism of asparagus bean in the future.

## Introduction

Soil salinity is among the leading environmental stresses affecting global agriculture, causing major reductions in crop productivity and quality [1]. This abiotic stressor is a growing problem, affecting approximately 20% of irrigated land and leading to continuing loss of arable areas in the world [2,3]. Salt stress inhibits the growth and productivity of crops via several distinct processes including photosynthesis limitation, metabolic dysfunction, and cellular structure damage [4]. Generally, there are at least two primary stresses on plants caused by salinity [5]. The first one is hyperosmotic stress resulting from the reduction of water potential and consequently diminished water availability. The second one is hyperionic stress caused by the toxic effects of accumulated ions. Salt-sensitive plants confine the absorption of salt and synthesize compatible solutes (e.g., proline, glycine betaine, and sugars) to adapt to their osmotic pressure. Salt-tolerant plants uptake and accumulate salt into the cell vacuoles, adjusting the salt concentrations of the cytosol and keeping a high cytosolic K^+^/Na^+^ concentration ratio in their cells [6–8]. Ion exclusion mechanisms could partly improve plant salt tolerance to relatively low concentrations of NaCl, but would not work at high concentrations of salt [9].

Salt tolerance is a complex trait in various plants and is usually regulated by multiple biosynthetic and signaling pathways [10]. Actually, with increasing investigation into plant salt tolerance, three main concepts of salinity tolerance mechanisms in crop plants have been described, including osmotic tolerance, ion exclusion, and tissue tolerance [11,12]. In maintenance of osmotic homeostasis, plants commonly sequester Na^+^ into the vacuole or accumulate compatible solutes by biosynthesis [13,14]. To regulate Na^+^efflux and vacuolar compartmentalization under salt stress, SOS1, a plasma membrane protein Na^+^/H^+^antiporter, was activated by interacting with the calcium sensor protein SOS3 and a Ser/Thr protein kinase SOS2 [13,15,16]. In the cytosol, a high K^+^/Na^+^concentration ratio was critical for cytoplasm ion balance during plant growth in high salinity soils [6]. Tissue tolerance is related to the ability of organs to function normally with high Na^+^ and Cl^-^ concentration in tissues or cells [12]. A number of component factors contribute to plant tissue tolerance, including transporting of Na^+^ and Cl^-^ and the maintenance of functional water status in leaves [12,17]. Moreover, plant salt tolerance appears to be a developmentally regulated process that differs depending on plant age and developmental stages [9, 18]. Recent advances have investigated salt-tolerance-related genes, such as *OsNHX2*, *GmsSOS1*, and *SOS2*, that helped to better understand the key components of the plant salt tolerance network [15,16,19]. However, large gaps exist in complete comprehension of the salt tolerance trait [20].

The past decade has seen the application of next-generation DNA sequencing technologies to the analysis of crops and model species under stress [21]. In these sequence-based profiling methods, the RNA-sequencing (RNA-seq) technique has been widely employed to investigate gene expression profiles of plants at different developmental stages in response to salt stress. By comparison with earlier methods of transcriptome sequencing, the RNA-seq technique has dramatically increased the throughput of RNA sequencing and allowed comprehensive measurement of transcript abundance [22].

Asparagus bean [*Vigna unguiculata* ssp. *sesquipedalis* (L.) Verdc], a member of the Fabaceae family, is among the top five edible legumes planted worldwide [23]. Currently, soil salinity represents one of the major constraints on the productivity of asparagus bean in agriculture. Morphological feature varied in different genotypes of *V*. *unguiculata* under salt stress [24–26]. Although great progress has been made on the genome sequencing of cowpea (*V*. *unguiculata*) in recent years [27,28], knowledge on the genetic basis of salt tolerance is still limited in asparagus bean (*V*. *unguiculata* ssp. *sesquipedalis*).

Therefore, the aim of this study was 1) to investigate whole-transcriptome expression profiles of transcripts through RNA-seq in asparagus bean under salinity conditions and 2) to explore differentially expressed genes (DEGs) related to salt stress. Two asparagus bean cultivars (one salt-sensitive genotype and one salt-tolerant genotype) were treated with NaCl (125 mM) at the seedling stage. A set of non-redundant transcripts have been generated, and then they have been used for the analysis of gene annotation, functional categorization and identification of candidate NaCl stress-responsive genes.

## Materials and methods

### Plant materials and growth conditions

Two asparagus bean cultivars with different salt tolerance were used in this study. One is salt-sensitive type (*V*. *unguiculata* ssp. *sesquipedalis* var. Zhijiang14, Coded “A10”) and the other is salt-tolerance type (*V*. *unguiculata* ssp. *sesquipedalis* var. Yulongteyou, Coded “A33”) [29]. Both were collected from the Hubei Province Engineering Research Center for Legume Plants, China.

The seeds of the two cultivars were sterilized with 70% ethanol for 1 minute, followed by washing with sterile water. Filled seeds of uniform size were sown in plastic pots containing the same soil matrix. The plastic pots were placed under long-day (14 h light /10 h dark cycle) conditions at temperatures of 30°C (light) and 20°C (dark) in an environmental chamber (HP1000GS-B, Wuhan Ruihua, China) with 60% relative humidity. After two weeks, seedlings at the same growth status were transplanted into standard Hoagland nutrient solution for three days to acclimate at a hydroponic culture condition. On the fourth day, two-week-old seedlings were transferred to the Hoagland solution with NaCl concentration of 125 mM. Meanwhile, control seedlings were treated in the Hoagland solution without NaCl (CK). Due to different salt tolerance mechanisms stimulated by salt stress [18], six time points (0 h, 6 h, 12 h, 24 h, 48 h, and 78 h) were set for the leaf and root sampling under salt stress treatment. The “0 h” point was set for control samples (CK). Three biological replicates of roots and leaves were randomly sampled at the five points after the treatment, and then they were mixed together as one sample. Each sample was immediately frozen in liquid nitrogen and stored at -80°C for future use. At last, roots and leaves of the two cultivars were harvested from all six time points (0 h, 6 h, 12 h, 24 h, 48 h and 78 h) for treatment and control. As a result, a total of 24 samples were prepared for RNA libraries.

### RNA extraction, library construction, and sequencing

The total RNAs were extracted from roots and leaves using Colum Plant RNA extraction Kit (BioTeke, Cat. No. RP3202) according to the manufacturer’s instructions. The RNA samples were digested using DNase I (RNase-free) (Fermentas, Cat. No. EN0521) at 37°C for 30 min to remove potential genomic DNA contamination. Then, the RNAs were examined by gel electrophoresis and quantified with NanoDrop (Quawell Q5000, Quawell Technology). Integrity of the quantified RNA samples was analyzed using an Agilent 2100 Bioanalyzer (Agilent Technologies, Palo Alto, CA, U.S.A). High-quality RNAs (RIN > 8) from each time point were used for cDNA preparation and RNA-seq.

The cDNA libraries were constructed using TruSeq RNA Sample Preparation V2 (Illumina, USA) according to the manufacturer’s instructions. Poly (A) mRNA was purified from total RNA using magnetic beads with oligo (dT) primers. The mRNA was then fragmented and purified with a high-temperature dilution after fragmentation. These cleaved mRNA fragments were then reverse transcribed into first strand cDNA using reverse transcriptase and random hexamers primers. Subsequently, RNA templates were removed, and the second strands were synthesized. The double-stranded cDNAs (ds cDNAs) were purified using AMPure XP beads. After purification, the ds cDNAs were subjected to end repair, single “A” nucleotides were added, and they were ligated to sequencing adapters. The products were then enriched by PCR with a cocktail primer to construct the cDNA library.

After quality control with Agilent 2100 Bioanalyzer and Qubit (Thermo Fisher Scientific) to detect fragment size and concentration, the libraries were sequenced using the Illumina HiSeq 2500 system at the Institute of Genetics and Developmental Biology, Chinese Academy of Sciences (Beijing, China).

### Transcript assembly and annotation

Raw sequencing image data were transformed into raw reads by base calling. The raw reads were filtered for clean reads by Trimmomatic [30]. The criteria was set as follows: adapter mismatch ≤ 2 bases; score of adapter palindrome mode match ≥ 30; score of adapter simple mode match ≥ 3; the adaptor leading and trailing ≥ 2 bases; the sliding window 1:2; the minimized reads length ≥ 50 bases. The subsequent analysis was based on these clean and high-quality reads. The clean reads from each library were *de novo* assembled into contigs with Trinity software [31]. The generated contigs were blasted to the NR database to remove the contaminating bacterial sequences. The clean contigs were blasted to get rid of high-similarity sequences and to obtain sequences that can no longer be extended on either end, which were referred as all-unigene. All-unigene sequences were aligned using BLASTX against the Nr, Swiss-Port, KEGG (Kyoto Encyclopedia of Genes and Genomes), and COG (Clusters of Orthologous) databases to determine or predict protein sequence with lengths ≥ 50 amino acids. All unigenes that could not be aligned to sequences in any of the databases mentioned above were translated into predicted protein with lengths ≥ 100 amino acid using the software Translation. For annotations, all unigenes were searched against the NR database. To obtain the GO (Gene Ontology) terms to describe biological process, molecular function and cellular components, Blast2GO software was used for the GO annotation, and the results were functionally classified by WEGO (Web Gene Ontology) at macromolecular level. BLASTX analysis against the KEGG pathway database was performed to assign putative metabolic pathways to all-unigene [32]. Additionally, the COG database was also used for further analysis of these unigenes.

### Identification of differentially expressed genes (DEGs)

DEGs under salt stress were analyzed. All the *de novo* clean reads were firstly assembled together to construct a reference genome, because the whole genome of *V*. *unguiculata* ssp. *sesquipedalis* is unavailable yet. Then each sample reads were aligned to the reference genome using the TopHat software 2.0.12 [33]. Subsequently, the TopHat-generated sequences were put into the software Cufflinks [34] and were run to calculate FPKM to indicate gene expression levels and significant differential expression between samples. Within each cultivar, DEGs were from the comparison between control (CK) and NaCl treatment during different treating time (6 h, 12 h, 24 h, 48 h, and 78 h). Between the two cultivars, DEGs were from the comparison at the same treating time and in the same tissue (root or leaf). The DEGs were defined as genes with FDR ≤ 0.001 and the absolute ratio of log_2_ (FPKM sample1/FPKM sample2) ≥ 1 set as threshold values.

The GO terms and the KEGG pathways enriched within DEGs were also analyzed with the Fisher’s exact test and the BH multiple testing correction method.

Hierarchical cluster analysis of the expression patterns of DEGs was performed after normalization by MultiExperiment Viewer v4.9 (The TM4 Software Development Team, http://mev.tm4.org/) [35]. The GO enrichment of DEGs was analyzed by the AgriGO [36]. The GO functional enrichment analysis was tested at a significance cutoff of 0.05 false discovery rate (FDR).

### Analysis of transcription factor families

Online software PlantTFcat (http://plantgrn.noble.org/PlantTFcat) [37] was employed to identify the potential genes of transcription factors (TFs) in the assembled unigene sequences derived from the salt-stress-induced RNA-seq data.

### qRT-PCR validation of differential expression genes

To validate the DEGs between the two asparagus bean cultivars, candidate genes were selected randomly for qRT-PCR tests (quantitative real-time RT-PCR) run on three independent biological samples of each time point. The total RNA was isolated using the same method described above. Reverse transcription reactions were performed using PrimeScript 1st strand cDNA Synthesis Kit (TAKARA). The primers were designed using an online primer tool (http://sg.idtdna.com/calc/analyzer) to amplify 100–250 bp regions of the selected genes. The following genes and primers were used as references: *gapdh* (F:5’ATCAGCCAAGGACTGGAGAG3’; R:5’ACGGAATGCCATACCAGTCA3’) (Tm = 62°C), *EF1a 2*α (F:5’ATCATCGTGGTTACTCCTTTAT3’; R:5’TCAGACTCTTCTTACCATCA3’) (Tm = 60°C), *EF1a 1α1* (F:5’GATTTCATGTAGCCGTAGCC3’; R:5’ATTTAAGACATCCCTCCTCAG3’) (Tm = 60°C).

Real-time PCR was performed on an ABI 7900HT PCR instrument in a 20 μL reaction volume containing 10 μL SYBR *Premix Ex Taq* II (Tli RNaseH Plus), 0.5 μL primer pairs (internal standard and target genes), and 2 μL (50 ng μL^−1^) cDNA. The reactions were carried out as follows: 95°C for 10 min followed by 35 cycles of 95°C for 15 s, 55°C for 60 s, and 72°C for 20 s. With the salt-sensitive asparagus bean as a control, the relative expression levels of chosen genes were normalized to the expression levels of reference genes calculated from cycle threshold values using the 2^−ΔΔCt^ method [38].

### Identification of microsatellites and primers calling

As byproducts, the assembled unigenes sequences were analyzed to identify potential microsatellites using software MISA (MIcroSAtellite) [39]. The criteria of microsatellites were set based on different repeat motif type. The minimum number of repeat motifs was set as following: mono-nucleotide repeats for ten, di-nucleotide repeats for six, tri-nucleotide repeats for five, tetra-nucleotide repeats for five, penta-nucleotide repeats for five and hexa-nucleotide repeats for five. Maximal number of bases interrupting two SSRs in a compound microsatellite was less than 100 base pairs. Microsatellite primers were designed using Primer 3 (http://primer3.sourceforge.net/).

### Mapping of the salt-stress-induced DEGs in asparagus bean genome

To identify the genomic location of the salt-stress-induced DEGs in asparagus bean, alignment between the DNA sequences of the DEGs and newly-published genome sequences of *Vigna unguiculata* v1.0 (GenBank accession: MATU00000000.1, https://legumeinfo.org/blat). Subsequently, SNP loci linked to the DEGs regions were revealed by alignment between the genome sequence *Vigna unguiculata v1*.*0* and a recently published SNP map of *V*. *unguiculata* [40]. After that, the genomic position of the DEGs and their adjacent SNP loci could be identified.

## Results

### Illumina sequencing, quality filtering, and *de novo* assembly

In this study, we constructed 24 cDNA libraries, including leaf and root samples from the two asparagus bean cultivars and six time points (0 h, 6 h, 12 h, 24 h, 48 h, and 78 h) for treatment and control. These libraries were then sequenced on the Illumina HiSeq 2500 platform. After removing sequencing adaptors and low quality data, 692,086,838 filtered reads were obtained amounting to 86.48 Gb of data (S1 Table). Transcriptome was *de novo* assembled using the Trinity software. Statistics of all the unigenes was listed in Table 1. 121,138 unigenes were generated, with a total length of 150,960,298 bp. The lengths of all-unigene ranged from 201 to 32,391 bp. The average unigene length was 968 bp with the N50 of 1,720 bp.

**Table 1:** Statistics analysis for all the unigenes assembly of asparagus bean.

Type	Number
The number of unigenes	121,138
The total length	150,960,298 (bp)
Minimum length	201 (bp)
Maximum length	32,391 (bp)
Average length	968 (bp)
N50	1,720 (bp)

### Functional annotation and classification of assembled transcripts

After assembly, all the 121,138 unigene sequences were searched against several public databases, including the NR, GO, COG, and KEGG databases using BLAST.

**Fig 1:**
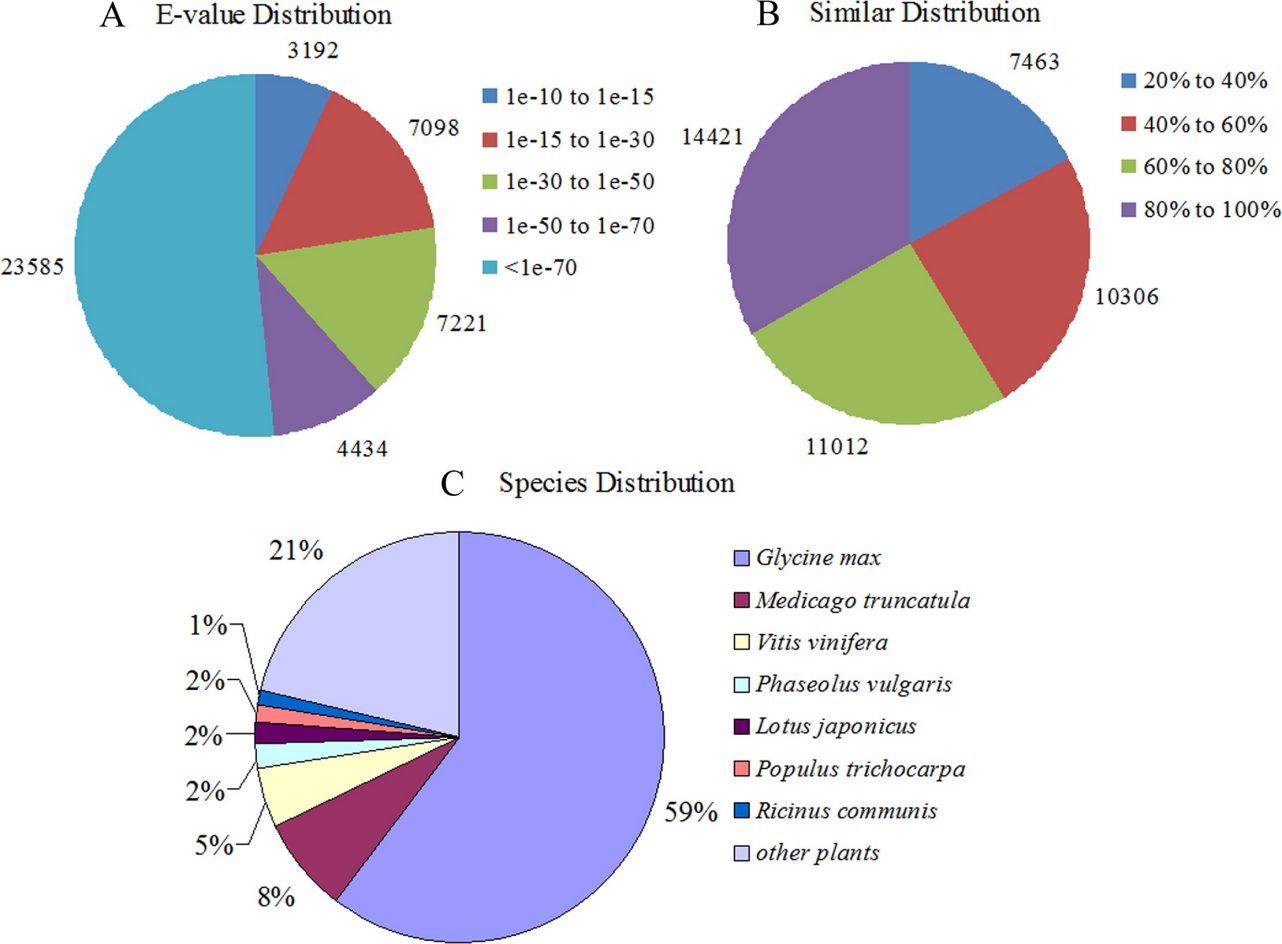
Results of similarity analyses of all unigenes against the NR database.

65,322 unigene sequences with each length less than 500 bp had BLASTX hits in the NR database (S2 Table). Among them, 28,019 unigene sequences were mapped to known genes with the best hit (E-value < 1e^-50^) (Fig 1A). Based on similarity distribution analysis, 14,421 unigene sequences matched deposited sequences with a similarity > 80% (Fig 1B). Moreover, 67% of the annotated unigenes could be assigned to the sequences from the top two legume species: *Glycine max* (59%) and *Medicago truncatula* (8%) (Fig 1C).

27,936 unigenes were annotated based on the GO analysis. Notably, biological regulation, cellular processes, metabolic processes, and response to stimulus were significantly over-represented in the 23 biological process GO groups. In the further analysis of GO classification, 27,794 unigenes was assigned to 25 COG categories (S3 Table). “General function prediction only” represented the largest group (5,681), followed by “post-translational modification, protein turnover, chaperones” (2,872).

14,068 unigenes were annotated in 125 KEGG pathways. “Metabolic pathways” was the largest group, followed by “ribosome”, and “biosynthesis of secondary metabolites” (S4 Table).

Additionally, based on PlantTFcat analysis, 98 TF families were identified from the assembled 121,138 unigenes. These TFs contained 4,524 unigenes, accounting for 3.7% of the total 121,138 unigenes. Of the 98 families, “C2H2” (655, 14.4%) was predominant, followed by “WD40-like” (401, 8.9%), “CCHC(Zn)” (348, 7.7%), “MYB-HB-like” (304, 6.7%), “AP2-EREBP” (194, 4.3%), “bHLH” (168, 3.7%), “PHD” (155, 3.4%), “Hap3/NF-YB”(138, 3.1%), “NAM” (127, 2.8%), “WRKY” (120, 2.7%), “bZIP”(105, 2.3%),”Homobox-WOX”(100, 2.2%), and “C3H” (96, 2.1%) (S5 Table).Therefore, the annotation of these salt-stress-induced unigenes gave us a global view of transcriptome in asparagus bean under salt stress.

### DEGs of asparagus bean in response to salt stress between the two cultivars

**Fig 2:**
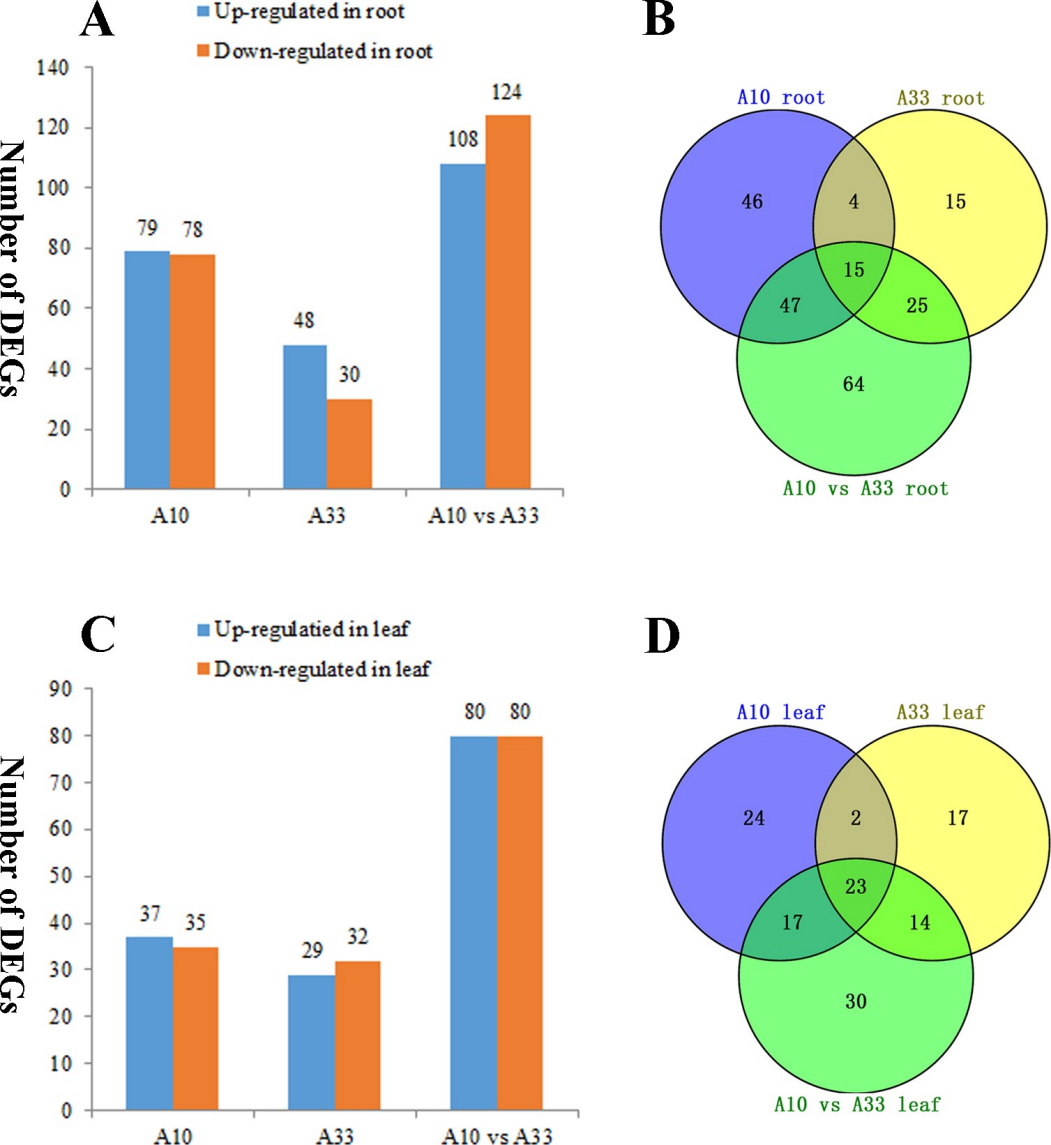
Overview of the 127 leaf-based DEGs and 216 root-based DEGs at the six time points (0 h, 6 h, 12 h, 24 h, 48 h and 78 h). (A) Numbers of DEGs compared between two samples in root at the six time points (0 h, 6 h, 12 h, 24 h, 48 h and 78 h). (B) Venn diagram showing the overlap of the DEGs at the six time points between “A10” root, “A33” root and “A10” vs “A33” root. (C) Numbers of the DEGs compared between two samples in leaf at the six time points (0 h, 6 h, 12 h, 24 h, 48 h and 78 h). (D) Venn diagram showing the overlap of the DEGs at the six time points between “A10” leaf, “A33” leaf and “A10” vs “A33” leaf. A10: sum of the DEGs in “A10-0h vs A10-6h”, “A10-0h vs A10-12h”, “A10-0h vs A10-24h”, “A10-0h vs A10-48h”, “A10-0h vs A10-78h”. A33: sum of the DEGs in “A33-0h vs A33-6h”, “A33-0h vs A33-12h”, “A33-0h vs A33-24h”, “A33-0h vs A33-48h”, “A33-0h vs A33-78h”. “A10 vs A33”: sum of the DEGs in “A10-0h vs A33-0h”, “A10-6h vs A33-6h”, “A10-12h vs A33-12h”, “A10-24h vs A33-24h”, “A10-48h vs A33-48h”, “A10-78h vs A33-78h”.

In this study, a set of salt-stress-induced DEGs were obtained (Fig 2; S6 Table). In the roots of the two cultivars, 467 DEGs were revealed including 235 upregulated DEGs and 232 downregulated DEGs. And 293 DEGs (146 upregulated DEGs and 147 downregulated DEGs) were found in their leaves (Fig 2A and 2C; S6 Table). After removing the repeated unigenes in all the DEGs, a total of 127 leaf-based DEGs and 216 root-based DEGs were identified between “A10” and “A33” (Fig 2B and 2D).

Compared with the salt-tolerance cultivar “A33”, the salt-sensitive cultivar “A10” triggered much more DEGs both in roots and in leaves. As shown in Fig 2A, regarding the number of up-regulated DEGs in roots, there was forty-eight DEGs in the “A33” and seventy-nine DEGs in the “A10”. Similarly, the number of down-regulated DEGs was thirty in the “A33” roots and seventy-eight in the “A10” roots.

**Fig 3:**
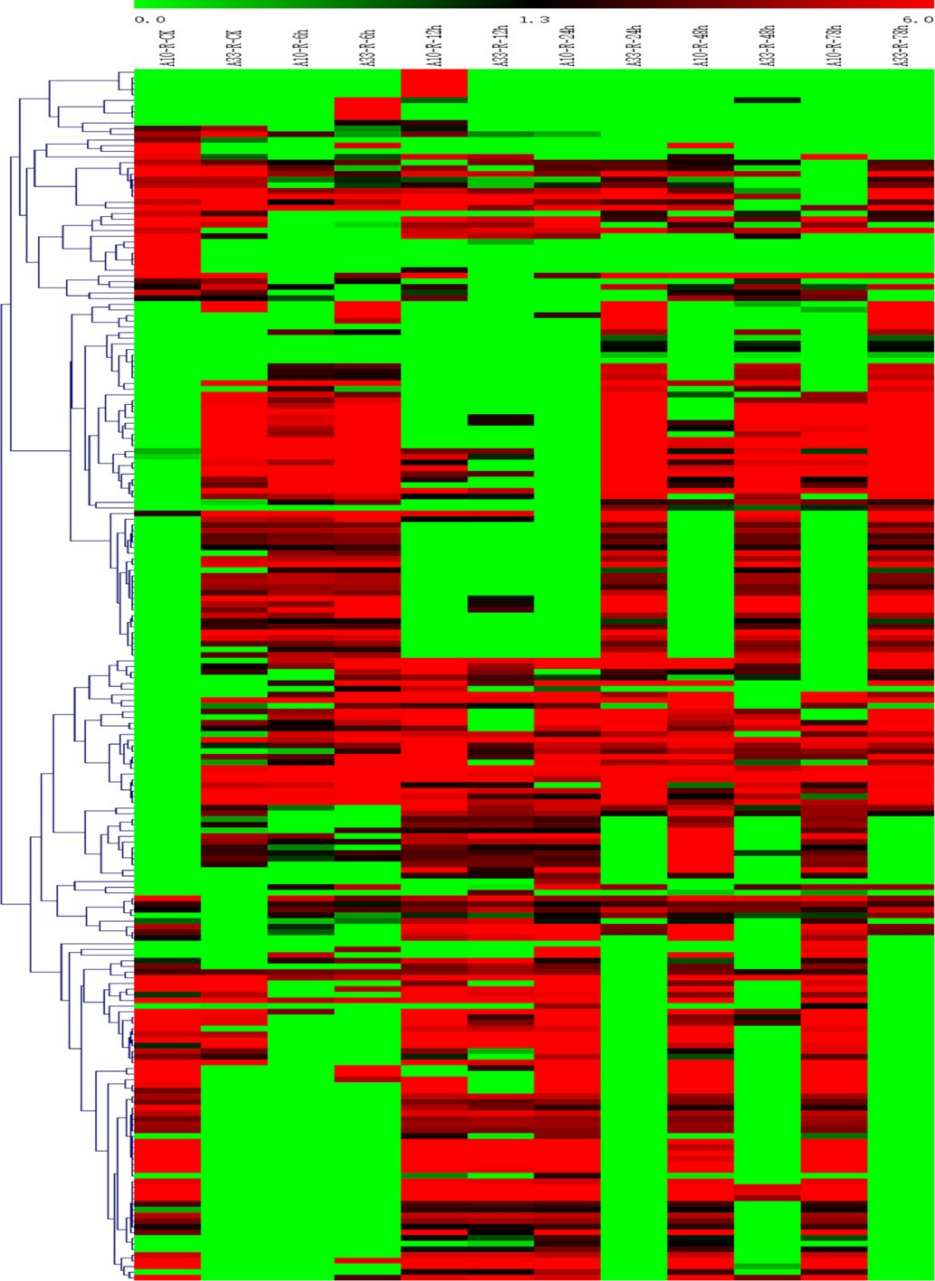
The Heatmap of the 216 root-based DEGs in roots of the two cultivars at all the six time points under salt treatment stages. R, root; L, leaf.

Furthermore, gene expression patterns of roots and leaves were analyzed between “A10” and “A33” under salt stress conditions. As shown in Fig 3, a heatmap of the 216 root-based DEGs exhibited temporal and spatial expression patterns between the two cultivars during salt stress treatment. These novelty DEGs would help to reveal the differentiation of salt tolerance occurred between the two cultivars under salt stress.

### Functional annotation of the leaf-based and root-based DEGs between the two cultivars

**Fig 4:**
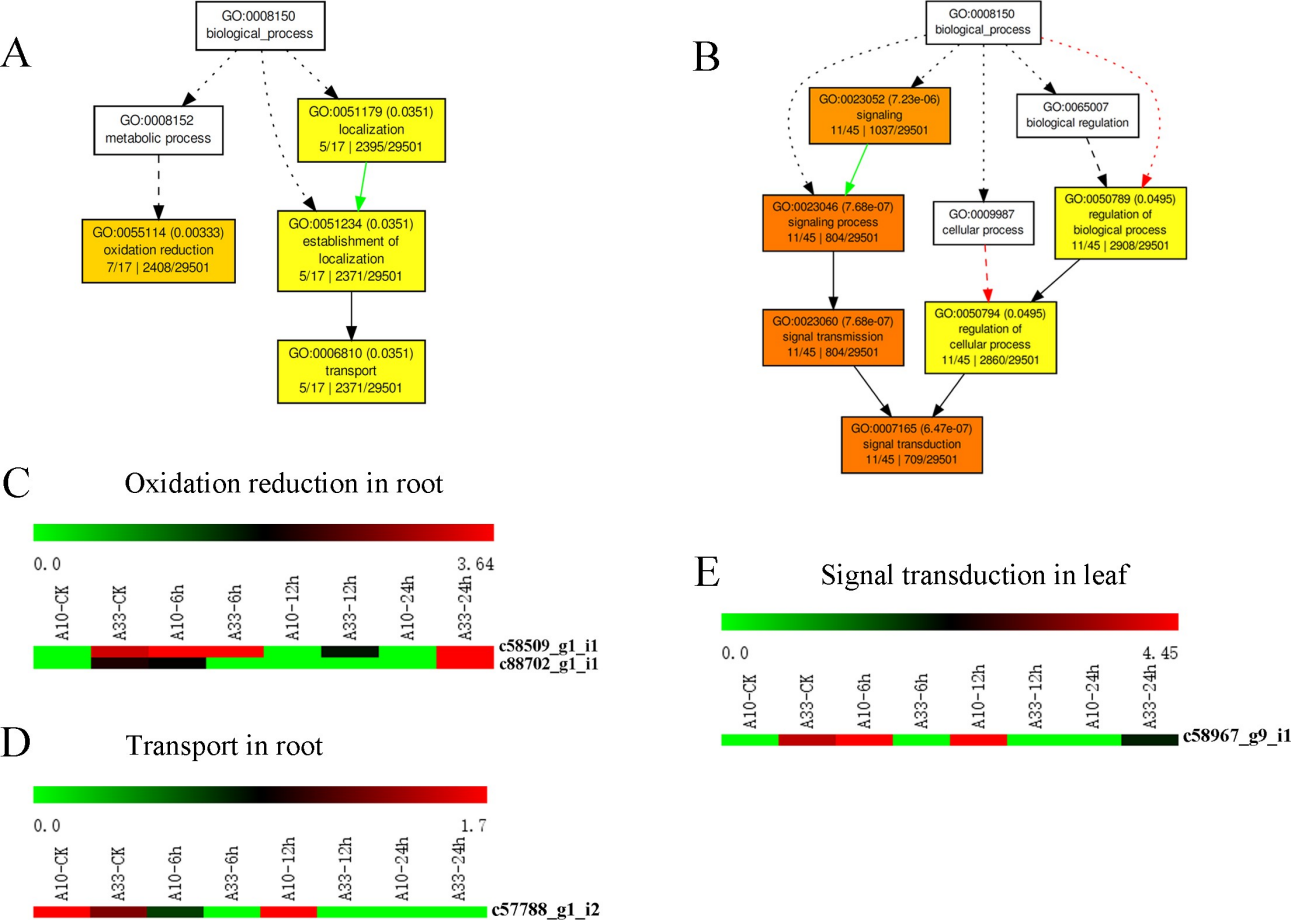
The GO enrichment of the four DEGs between “A10” and “A33” at CK (0h) and at the salt stress treatment stages (6 h, 12 h, and 24 h). (A) The GO enrichment of the DEGs between the “A10” root and the “A33” root at CK (0 h) and at the salt stress treatment stages (6 h, 12 h, and 24 h). (B) The GO enrichment of the DEGs between the “A10” leaf and the “A33” leaf at CK (0 h) and at the salt stress treatment stages (6 h, 12 h, and 24 h). (C) and (D) The heatmaps of the DEGs of enriched GO terms between the “A10” root and the “A33” root at CK (0 h) and at the salt stress treatment stages (6 h, 12 h, and 24 h). (E) The heatmaps of DEGs of enriched GO terms between the “A10” leaf and the “A33” leaf at CK (0 h) and at the salt stress treatment stages (6 h, 12 h, and 24 h).

The DEGs were functionally annotated based on GO enrichment analysis and were sorted into different “biological process” categories. All of the 127 leaf-based DEGs and the 216 root-based DEGs were aligned against the GO database (Fig 4A and 4B). Results showed that four DEGs (c58509_g1_i1, c88702_g1_i1, c57788_g1_i2, and c58967_g9_i1) might participate in the biological processes of “oxidation reduction”, “localization”, and “signal transduction”. Three of the four DEGs expressed in root and the rest one was found in leaf. The three root-based DEGs were involved in the process of “oxidation reduction” (c58509_g1_i1, c88702_g1_i1) (Fig 4C) and “transport” (c57788_g1_i2) (Fig 4D). The one leaf-based DEG was involved in “signal transduction” (c58967_g9_i1) (Fig 4E). These four DEGs provide a valuable resource for investigating salt-stress-related biological processes, functions, and pathways in asparagus bean.

### Identification of salt-stress-induced transcription factor families in the DEGs

**Fig 5:**
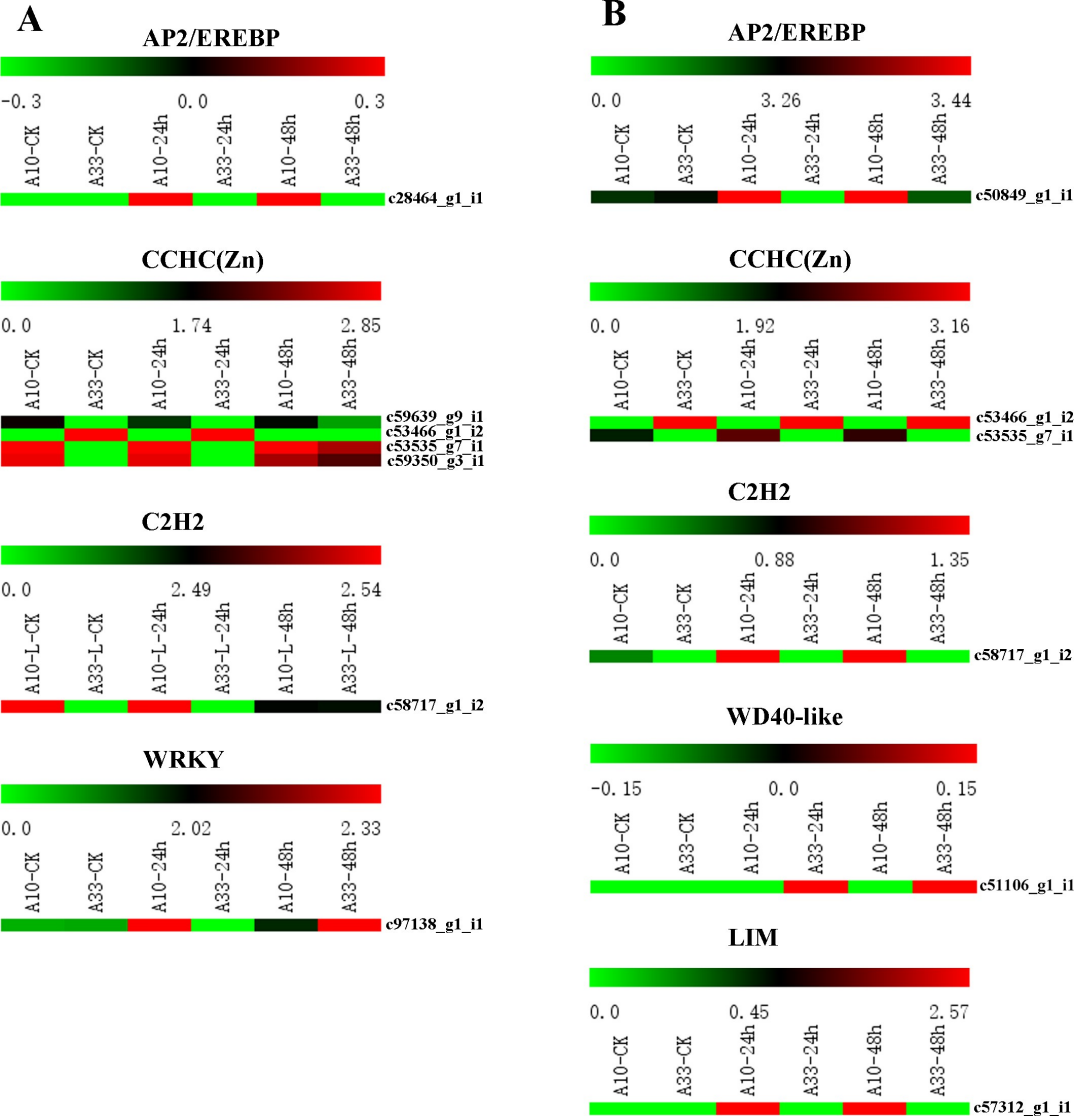
The heatmaps of the thirteen DEG-related TFs between “A10” and “A33”. (A) The DEG-related TFs in leaf at CK (0 h) and the salt stress treatment stages (24 h, and 48 h). (B) The DEG-related TFs in root at CK (0 h) and the salt stress treatment stages (24 h, and 48 h).

Plants initiate transcription factors to regulate expression of genes in plant development and stress responses. Our results indicated that thirteen DEGs from the 127 leaf-based and 216 root-based DEGs were assigned to different TF families. In the leaf-based DEGs, seven were classified into four TF families including the AP2/EREBP (c28464_g1_i1), CCHC(Zn) (c59639_g9_i1, c53466_g1_i2, c53535_g7_i1 and c59350_g3_i1), C2H2 (c58717_g1_i2), and WRKY (c97138_g1_i1) families (Fig 5A). In the root-based DEGs, six were clustered into five TF families, including the AP2/EREBP (c50849_g1_i1), C2H2 (c58717_g1_i2), CCHC(Zn) (c53466_g1_i2, and c53535_g7_i1), WD40-like (c51106_g1_i1) and LIM (c57312_g1_i1) families (Fig 5B).

Among the thirteen TFs, three TFs ((CCHC(Zn) (c53466_g1_i2), CCHC(Zn) (c53535_g7_i1) and C2H2 (c58717_g1_i2) illustrated opposite regulation under salt stress between the two cultivars. As can be seen from the Fig 5B, c53535_g7_i1 and c58717_g1_i2 showed up-regulation in both roots and leaves of “A10”. On contrary, both of them were down-regulated in both roots and leaves of “A33”. Similarly, c53466_g1_i2 was down-regulated in “A10”, but it was up-regulated in “A33”. Thus, these TF-related DEGs would play diverse roles in salt stress response and adaptation in asparagus bean.

### Validation of salt-stress-induced DEGs by qRT-PCR between the two cultivars

**Fig 6:**
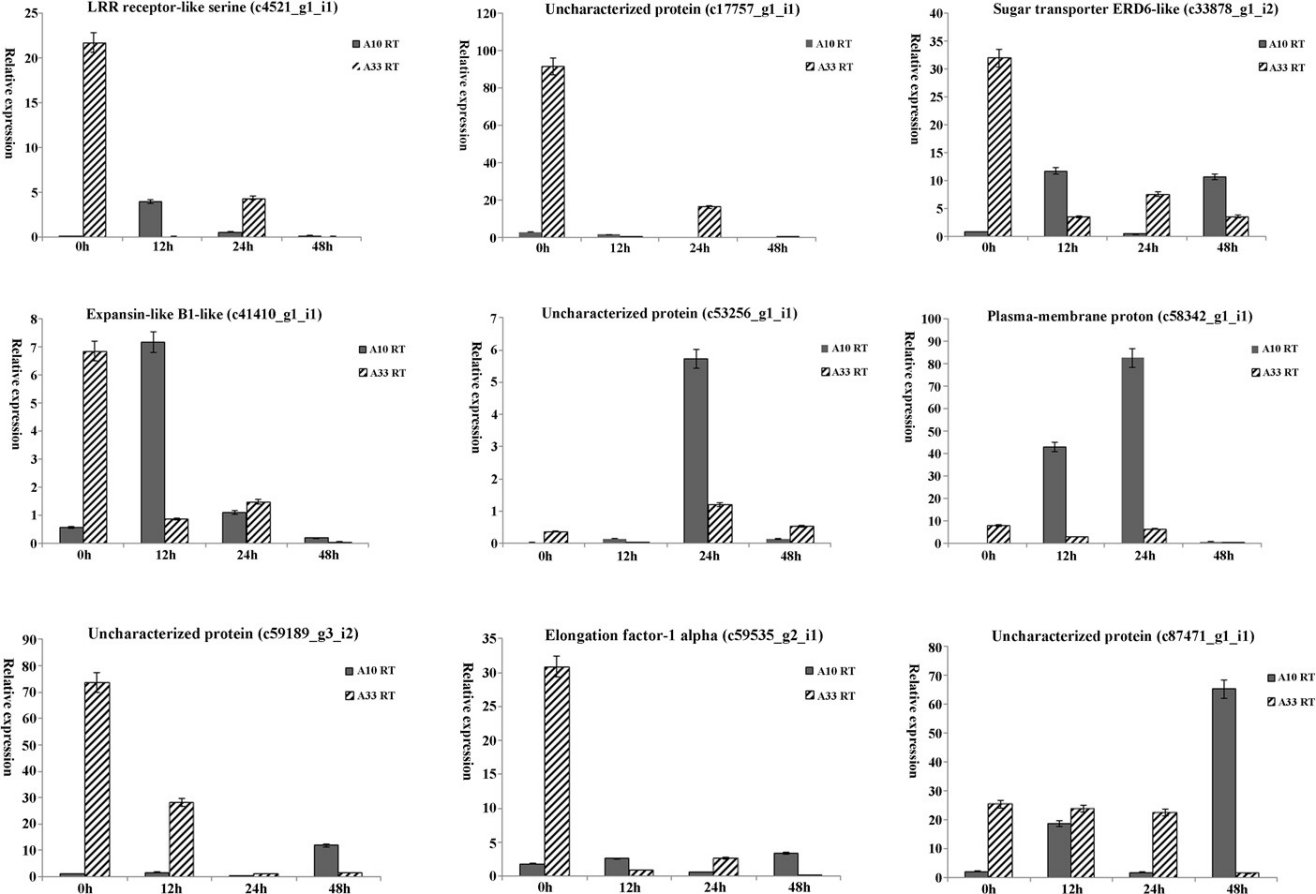
qRT-PCR analyses of nine salt-stress-induced DEGs in asparagus bean under salt stress.

To validate the RNA sequencing results and expression profiling, nine salt-stress-induced DEGs were randomly selected for qRT-PCR analysis (Table 2). The root tissues of the two cultivars (“A10” and “A33”) were sampled for 0, 12, 24, and 48 h under salt stress and control. The qRT-PCR results demonstrated that the nine DEGs were salinity responsive. And most of them exhibited different responses to salt stress in both cultivars “A10” and “A33” (Fig 6). During the NaCl treatment, eight of the nine DEGs (c4521_g1_i1, c33878_g1_i2, c41410_g1_i1, c53256_g1_i1, c58342_g1_i1, c59189_g3_i2, c59535_g2_i1, c87471_g1_i1) were up-regulated in the “A10” roots but down-regulated in the “A33” roots. Only one exception is the DEG “c17757_g1_i1” which was down-regulated in both the “A10” and “A33” roots.

**Table 2:** Information of nine salt-induced DEGs in asparagus bean.

Gene name	Primer (5’-3’)	Tm (°C)	GO annotation/ GO term	GO ID
c4521_g1_i1	F:AGAAACAATGGCTGAGGC R:AACAACAAGGTCGCAAGG	57	LRR receptor-like serine/ regulation of cellular process	GO:0050794
c17757_g1_i1	F:TAGCAACCCAGAAGAAGGAATC R:TTCGCCGCCGAGTTATACT	54	uncharacterized protein/ response to osmotic stress	GO:0006970
c33878_g1_i2	F:AAGAGCCCTTCATTCAACC R:CCACAAACAGCAACAAGTG	57	sugar transporter ERD6-like/ transmembrane transporter	GO:0022891
c41410_g1_i1	F:AACACCACATTGTAGCCAGAAT R: GGAGACAGAACAGACTTCATCA	55	expansin-like B1-like/ sexual reproduction	GO:0019953
c53256_g1_i1	F:TTCTTCTGCTCATCAACGG R:CCACACCTACACAAACCAAC	57	uncharacterized protein/ response to biotic stimulus	GO:0009607
c58342_g1_i1	F:CAGAGATGGAGTGGATGTC R:GCACGGCTTCTTCTTCAT	50	plasma-membrane proton	GO:0006812
c59189_g3_i2	F:GAGTTCGTTGTAGAAAGTGTGG R:GCTCAATCAAAGAGAGGTATCC	57	uncharacterized protein/ cytoplasm	GO:0005737
c59535_g2_i1	F:CGACCAAGAGGAGGATAAGC R: CCAGTTGTTGATTGCCACAC	60	elongation factor-1 alpha/ cytoplasm	GO:0005737
c87471_g1_i1	F:CTTGAGATTAGCCGTGACGAAT R:TCACTCCTCACATTCTCTTCCA	55	uncharacterized protein phosphorelay signal transduction	GO:0000160

Moreover, the nine DEGs were predicted to have distinct functions, such as cation transport (c58342_g1_i1), response to osmotic stress (c17757_g1_i1), response to biotic stimulus (c53256_g1_i1), regulation of cellular process (c4521_g1_i1, c41410_g1_i1), substrate-specific transmembrane transporter activity (c33878_g1_i2), cytoplasm (c59189_g3_i2, c59535_g2_i1), and phosphorelay signal transduction system (c87471_g1_i1) (Table 2). Therefore, the nine salt-stress-induced DEGs could contribute to the difference in salt tolerance between the two cultivars “A10” and “A33”.

### The vesicular-transport-related KEGG pathway under salt stress in asparagus bean

**Fig 7:**
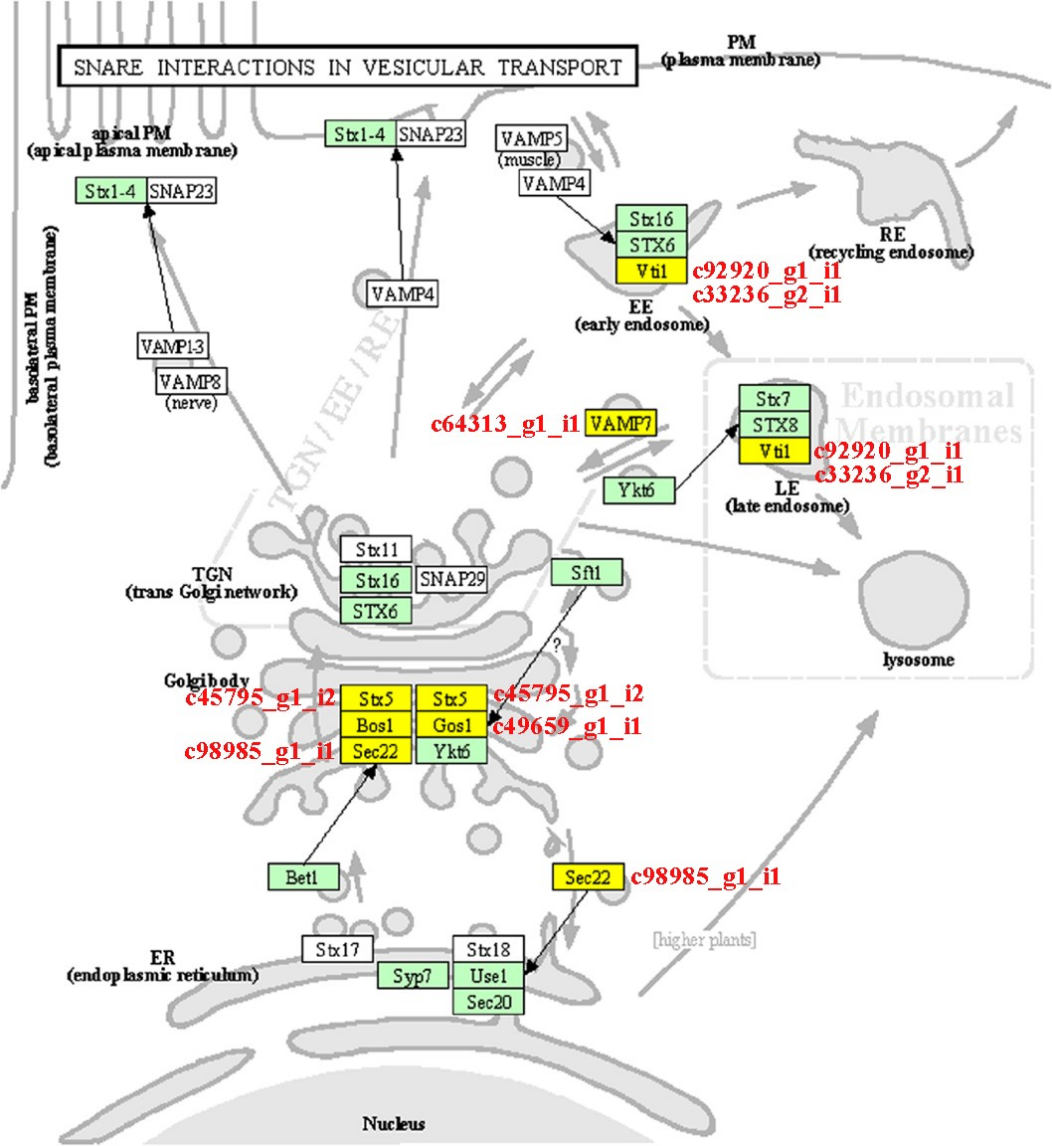
Schematic of the pathway category “SNARE interactions in vesicular transport” (ko:vvi04130). The map is from the KEGG database (http://www.genome.jp/kegg/).

Further analysis of these DEGs was conducted to investigate the biological functions in the 125 KEGG pathways (S7 Table). In the present study, notably, the pathway “SNARE interactions in vesicular transport” (ko:vvi04130), which was a salt-related process, was enriched. Six genes (c92920_g1_i1, c64313_g1_i1, c45795_g1_i2, c49659_g1_i1, c98985_g1_i1, c33236_g2_i1) were involved in “SNARE interactions in vesicular transport” (Fig 7). Thus, the “SNARE interactions in vesicular transport” pathway might be affected under salt stress conditions, implying a potential contribution to the regulation of salt stress in asparagus bean.

### Identification of microsatellites

Using MISA software, microsatellites were unveiled from the salt-stress-induced unigenes sequences in asparagus bean. By screening the 121,138 unigenes sequences, 24,417 potential microsatellites were identified from 19,805 microsatellites-containing sequences. Among the microsatellite sequences, there were 3,616 sequences containing more than one microsatellite, while 1,146 sequences presenting in compound formation (S8 Table). The most dominant microsatellite repeat type was mononucleotide repeats (15,388, 63.0%), followed by di-nucleotide (4,561, 18.7%), tri-nucleotide (4,182, 17.1%), tetra-nucleotide (237, 0.97%), penta-nucleotide repeats (25, 0.10%), and hexa-nucleotide (24, 0.10%) (S9 Table). Further, the predominant microsatellite motif was A/T (15341, 62.8%) among all the microsatellites. For di-nucleotide motifs, AG/CT showed the higher ratio (2193, 9.0%) followed by AT/AT (1,389, 5.68%), and AC/GT (975, 3.99%). In tri-nucleotide motifs, AAG/CTT exhibited the most abundant type (1,179, 4.83%), followed by AAT/ATT (724, 2.97%), ATC/ATG (690, 2.83%), ACC/GGT (429, 1.76%), and AAC/GTT (404, 1.65%) (S10 Table). 15, 321 microsatellite primers were designed based on those microsatellites (S11 Table). These primers would be useful for genetic analysis in asparagus bean.

### Location of the nine DEGs in asparagus bean genome

**Fig 8:**
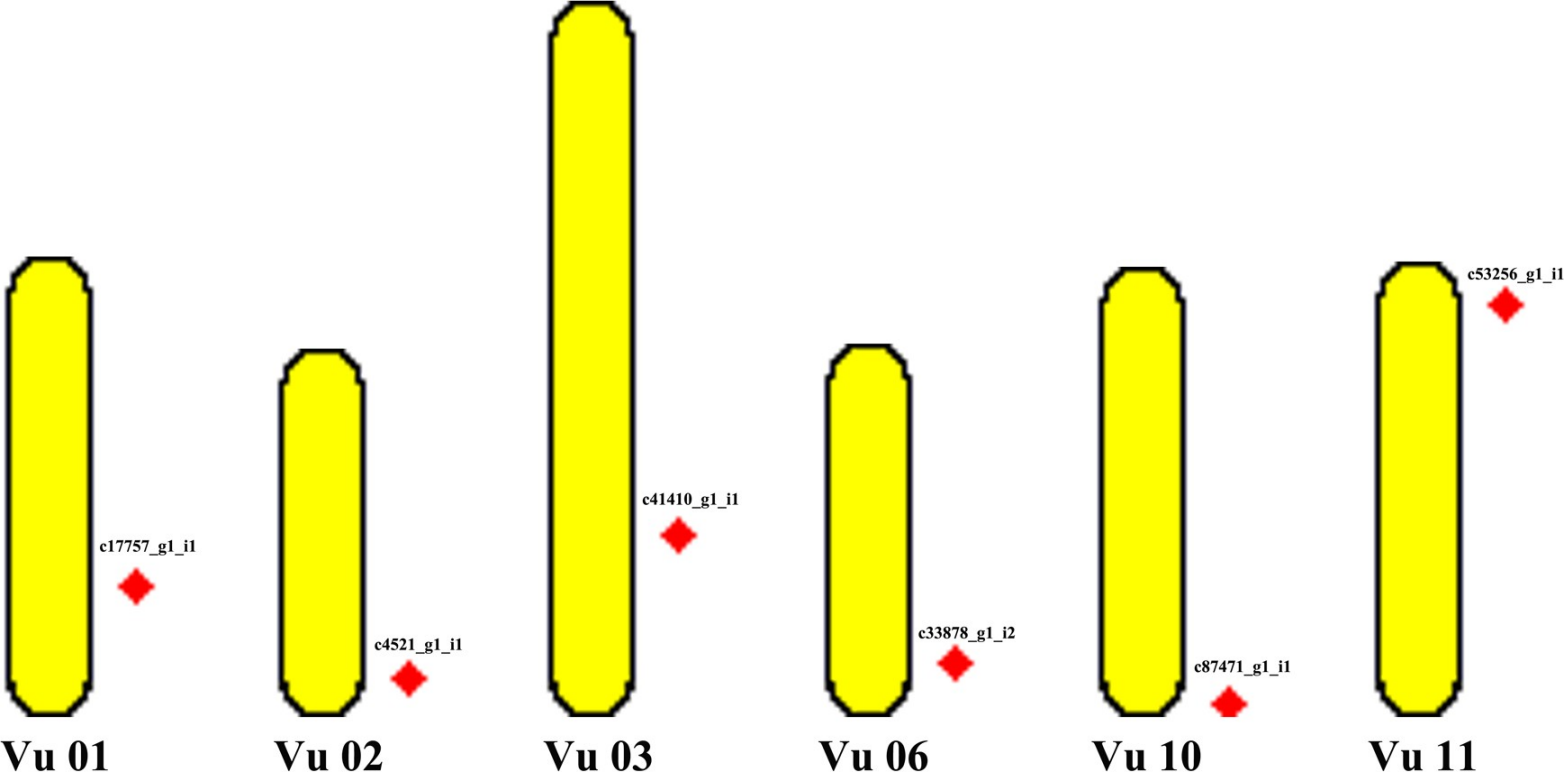
Mapping of six of nine salt-stress induced DEGs in asparagus bean genome.

Based on mapping analysis, only six of the nine salt-stress-induced DEGs were revealed to locate in different regions in the asparagus bean genome (Fig 8). Three DEGs (c58342_g1_i1, c59189_g3_i2, c59535_g2_i1) were unplaced in the asparagus bean genome. The other six mapped DEGs were “c4521_g1_i1”, “c17757_g1_i1”, “c33878_g1_i2”, “c41410_g1_i1”, “c53256_g1_i1”, and “c87471_g1_i1”. They distribute among six chromosomes in asparagus bean genome, including Vu 01 (“c17757_g1_i1”), Vu 02 (“c4521_g1_i1”), Vu 03 (“c41410_g1_i1”), Vu 06 (“c33878_g1_i2”), Vu 10 (“c87471_g1_i1”), and Vu 11 (“c53256_g1_i1”).

Further analysis found 17 SNP loci adjacent to the six DEGs (Table 3). There are five SNP loci (M12561, M29324, M29326, M29325, M29323) linked to “c4521_g1_i1”, four SNP loci (M28659, M14926, M28295, M28296) linked to “c53256_g1_i1”, three SNP loci (M10308, M21200, M21201) linked to “c17757_g1_i1”, two SNP loci (M18651, M6637) linked to “c33878_g1_i2”, two SNP loci (M9570, M29874) linked to “c41410_g1_i1”, one SNP locus (M7035) linked to “c87471_g1_i1”.

**Table 3:** Genomic location of the six salt-induced DEGs in asparagus bean genome.

Gene ID	Location ^Δ^	Chromosome	SNP ^#^	LG ^#^
c4521_g1_i1	Vigun02g154800	Vu02	M12561, M29324, M29326, M29325, M29323	LG6
c17757_g1_i1	Vigun01g124200	Vu01	M10308, M21200, M21201	LG3
c33878_g1_i2	Vigun06g173000	Vu06	M18651, M6637	LG8
c41410_g1_i1	Vigun03g298100	Vu03	M9570, M29874	LG4
c53256_g1_i1	Vigun11g030500	Vu11	M28659, M14926, M28295, M28296	LG6
c87471_g1_i1	Vigun10g179400	Vu10	M7035	LG1

Δ Data source from the website https://legumeinfo.org/blat

# Pan et al., 2017. Frontiers in Plant Science, 8, p.1544

The position of these DEGs and their SNPs was newly found, and they were not reported before. So, they provide new sight into asparagus bean genome concerning salt-stress-induced genes.

## Discussion

### Functional annotation, classification of assembled unigenes

In the present study, our results increased the number of transcript sequences available that could be integrated to public genome resources of asparagus bean [27, 28, 41–43].We identified 121,138 unigenes with an average length of 968 bp and N50 length of 1,720 bp. Based on GO analysis, there was 23 biological process groups. The dominant GO terms of the unigenes under salt stress concurred with previous similar studies, comprising of “cellular process” and “metabolic process” [44, 45]. A set of 343 DEGs (127 leaf-based DEGs and 216 root-based DEGs) were identified in asparagus bean seedlings between control and salt-treated conditions.

### The salt-stress-induced DEGs in the processes of “oxidation reduction”, “transport” and “signal transduction”

In higher plants, intracellular and extracellular antioxidants could give rise to complicated networks to protect against oxidation activities under abiotic stresses [46]. In this study, four novel DEGs were uncovered by the GO analysis. Three of them (c58509_g1_i1, c88702_g1_i1, and c57788_g1_i2) were in the root, while one of them (c58967_g9_i1) was in the leaf. They were predicted function in “oxidation reduction” (c58509_g1_i1, c88702_g1_i1) (Fig 4C) “transport” (c57788_g1_i2) (Fig 4D) and “signal transduction” (c58967_g9_i1) (Fig 4E). In the salt-tolerance genotype “A33”, the two genes (c57788_g1_i2, c88702_g1_i1) were down-regulated.

Generally, root is the plant organ that first senses and reacts to environmental stresses [47], and salt stress suppressed root growth in most plants [48]. Notably, in the oxidation reduction, gibberellin 2-oxidase (c58509_g1_i1) gene of the gibberellin catabolic process was up-regulated in the roots of “A33” under salt stress in this study. Gibberellin 2-oxidase gene encodes 2-oxoglutarate-dependent dioxygenase (2ODD), which changes bioactive and intermediate forms of GA to inactive forms [49]. Endogenous GA levels in *Arabidopsis* was decreased by the induction of GA 2-oxidase, causing that growth is repressed for high-salinity stress [50]. Recent results suggest that GA 2-oxidase is crucial for controlling Arabidopsis root meristem cell number and suppresses IAA-directed primary root and root hair growth in response to salt stress [51,52]. Similarly, the candidate gene of gibberellin 2-oxidase (c58509_g1_i1) possibly stimulate the oxidation reduction of asparagus bean in response to salt stress.

One DEG “c57788_g1_i2” was a candidate gene of transmembrane transporters NRT2 (nitrate transporter 2). The NRT2 family, which contains seven genes in *Arabidopsis*, encodes high-affinity NO^3-^ transporters in roots [53]. Except for nitrate availability, the NRT2 genes could be regulated at the transcriptional level by other stress factors [54–56]. In our study, the DEG “c57788_g1_i2” was down-regulated in the salt-tolerance genotype “A33”. As a result, its low expression level possibly affected root NO^3−^ uptake in asparagus bean, and finally caused growth reduction under low and limiting NO^3−^ availability.

In addition, a potential candidate gene (c58967_g9_i1), encoding protein suppressor of NPR1 (nonexpressor of pathogenesis-related protein 1), was related to signal transduction. Actually, the function of NPR1 is a crucial modulator of the plant UPR (unfolded protein response) that accompanies with ER stress-induced reduction of the cytosol and translocation of NPR1 to the nucleus [57]. Under salt stress, NPR1 might regulate SA signaling to mediate Na^+^ entry into roots and its subsequent transport into shoots [58]. So, this might be the reason that the DEG “c58967_g9_i1” showed up-regulation in the salt-sensitive genotype “A10” under salt stress. Undoubtedly, signal transduction system is pivotal for asparagus bean in defense responses against salinity.

### Novel TF-related DEGs under salt stress revealed in asparagus bean

Transcription factors regulated expression of salt-related genes and ultimately determined the level of salt tolerance of plants [3]. Many of these transcription factors relating to abiotic stress were predominant, including “WD40-like” [59], “CCHC(Zn)” [60], “AP2/EREBP” [61], “basic helix-loop-helix (bHLH)” [62], “WRKY” [63], and “bZIP” [64, 65]. Similar transcription factors were found and they exhibited different expression regulation in asparagus bean in this study. Four TFs (AP2/EREBP, CCHC(Zn), C2H2 and WRKY) in the leaf-based DEGs and five TFs (AP2/EREBP, C2H2, CCHC(Zn), WD40-like and LIM) were in the root-based DEGs. It is noticed that gene expression were up-regulated in four TFs (AP2/EREBP, CCHC(Zn), C2H2, and LIM) in the roots of salt-sensitive genotype “A10”. They may play an important role in coping with salt stress in asparagus bean.

As for AP2/EREBP, the increased level of expression of AP2/EREBP improves salt tolerance in *Oryza sativa* and *Arabidopsis* [66]. C2H2-type zinc fingers contribute to the potato response to salt and dehydration stresses through an ABA-dependent pathway [67]. C2H2 zinc-finger genes probably responded positively to salt stress and were upregulated in leaves and/or roots in poplar [68, 69]. In *Arabidopsis* C2H2-type zinc-finger proteins functioned as transcription repressors under salt stress conditions [70, 71]. During the salt stress process in *Arabidopsis*, overexpression of the CSDP1 gene (containing seven CCHC-type zinc fingers) suppressed seed germination, while overexpression of the CSDP2 (containing two CCHC-type zinc fingers) gene promoted seed germination [72]. In tomato, a novel regulatory gene, SlZF3, encodes a C2H2 zinc-finger protein and enhances plant salt-stress tolerance by interacting with CSN5B [73]. In wheat, two proteins (TaRZ1 and TaRZ2) containing a CCHC-type zinc finger were inhibited under salt stress conditions [74]. These novel TFs provided potential candidate genes for future understanding of the molecular mechanism of salt tolerance in asparagus bean.

### DEGs involved in ion transport, hormones, and signal transduction

The processes of ion transport, hormones, and signal transduction have important roles in response to salinity stress in asparagus bean. The nine tested DEGs are mainly involved in those processes. Several DEGs had potential for maintaining homeostasis in the ion transport process. Their function was associated with ion transmembrane transport activities, cation transport (c58342_g1_i1), substrate-specific transmembrane transporter activity (c33878_g1_i2), response to osmotic stress (c17757_g1_i1), and cytoplasmic localization (c59189_g3_i2, c59535_g2_i1). Another DEG (c58509_g1_i1) was related to the gibberellin catabolic process in asparagus bean. Previous studies showed that GA enhanced by salt stress, resulting in the irregular root growth [75, 76]. Therefore, to cope with salt stress, it is essential to activate plant hormone metabolism pathways in asparagus bean. Additionally, one of the DEGs (c87471_g1_i1), down-regulated in “A33”, was probably involved in the phosphorelay signal transduction system in asparagus bean under salt stress. Salt stress could stimulate the phosphorylation of the *Arabidopsis* vacuolar K^+^ Channel TPK1 by calcium-dependent protein kinases [77]. These novel DEGs might be the reason for the discrepancy of salt tolerance in asparagus bean.

### Mechanism of the vesicular-transport-related KEGG pathway under salt stress

Intracellular membrane dynamics play a significant role in plant salt tolerance [78]. In the KEGG pathway analysis, it is noticed that several DEGs were assigned to the pathway “SNARE interactions in vesicular transport” which mediates vesicular trafficking. Many researchers have studied SNARE complexes in plant tolerance to salt stresses [79–85]. The AtVAMP7C family of vesicle soluble *N*-ethylmaleimide-sensitive factor attachment protein receptors (v-SNAREs) mediates fusion of H_2_O_2_-containing vesicles with the tonoplast. Suppression of the *AtVAMP7C* genes that showed downregulation effect could improve plant salt tolerance [86]. Similarly, overexpression of *GsVAMP72*, which is a member of R-SNARE family in *Glycine soja*, resulted in the reduction of plant tolerance to salt stress [83]. Additionally, a Qc-SNARE protein (AtSFT12) took part in salt response via sequestration of Na^+^ in vacuoles [84]. In a word, as an important strategy of osmotic tolerance, this “SNARE interactions in vesicular transport” pathway influenced ion influx or efflux. It should play an important role in cellular metabolic processes, ultimately leading to the salt tolerance disparity among various asparagus bean cultivars.

### Molecular markers and mapping of the salt-stress-induced DEGs in asparagus bean

Molecular markers have been playing an increasing role in plant genetics studies. As byproducts in this study, a great number of microsatellites were screened out from sequences of all the unigene. Further, 15, 321 novelty microsatellite primers were obtained. Once the marker validation of these microsatellites is verified, they could be useful tools for future studies on genome mapping, marker-assisted selection, and population genetic analysis in asparagus bean. In addition, mapping of six salt-stress-induced DEGs exhibited their distribution among six chromosomes in asparagus bean genome. 17 SNP loci are linked to the six salt-stress-induced DEGs, which are different from the seven SNP markers related to salt tolerance at seedling stage in *V*. *unguiculata* [87]. These salt-stress-related markers (both microsatellites and SNP markers) would offer paths to shed light on the molecular genetic basis of salt tolerance in asparagus bean.

## Conclusion

In this study, a salt-stress-induced transcriptome of asparagus bean was obtained and annotated. A set of 343 DEGs (127 leaf-based DEGs and 216 root-based DEGs) were identified at seedlings of asparagus bean between control and salt-treated conditions. Four DEGs might be involved in the biological processes of “oxidation reduction”, “transport” and “signal transduction”. Seven leaf-based DEGs were assigned to four TF families (AP2/EREBP, CCHC(Zn), C2H2, and WRKY), while six root-based DEGs were found in five TF families (AP2/EREBP, C2H2, CCHC(Zn), WD40-like and LIM). Expression profiles of nine salt-stress-induced DEGs which involved in ion transport, hormones, and signal transduction were validated by qRT-PCR. In the KEGG pathway analysis, six genes were enriched in the salt-stress-related pathway “SNARE interactions in vesicular transport”. In addition, 15, 321 microsatellite markers were found in all the unigenes, while 17 SNP linked to six salt-stress induced DEGs were revealed. These candidate genes provide novel insights for understanding the salt tolerance mechanism of asparagus bean in the future.

## Supporting information

S1 Table Statistics of raw data for the salt-stress-induced asparagus bean transcriptome. (XLS)

S2 Table Statistical summary of sequencing and assembly results. (XLS)

S3 Table The COG classification for all the unigenes. (XLS)

S4 Table 125 KEGG pathway annotation. (XLS)

S5 Table Prediction of the transcription factor families in asparagus bean. (XLS)

S6 Table Summary of DEGs in the root and the leaf tissue between the salt-tolerant and salt-sensitive genotypes of asparagus bean. (XLS)

S7 Table All the unigenes in the125 KEGG pathways. (XLS)

S8 Table Results of microsatellite search in *V. unguiculata* ssp. *Sesquipedalis*. (XLS)

S9 Table Distribution to different repeat type classes. (XLS)

S10 Table Summary of the frequency for different microsatellites in *V. unguiculata* ssp. *sesquipedalis*. (XLS)

S11 Table The microsatellite primers in *V. unguiculata* ssp. *sesquipedalis*. (XLSX)

## References

[1] Wang X, Fan P, Song H, Chen X, Li X, Li Y. Comparative proteomic analysis of differentially expressed proteins in shoots of *Salicornia europaea* under different salinity. J. Proteome Res. 2009; 8: 3331–3345. 10.1021/pr801083a 19445527

[2] Yeo AR. Predicting the interaction between the effects of salinity and climate change on crop plants. Sci. Hortic. 1998; 78: 159–174. 10.1016/S0304-4238(98)00193-9

[3] Deinlein U, Stephan AB, Horie T, Luo W, Xu G, Schroeder JI. Plant salt-tolerance mechanisms. Trends Plant Sci. 2014; 19: 371–379. 10.1016/j.tplants.2014.02.001 24630845 PMC4041829

[4] Zhang F, Zhu G, Du L, Shang X, Cheng C, Yang B, et al Genetic regulation of salt stress tolerance revealed by RNA-Seq in cotton diploid wild species, *Gossypium davidsonii*. Sci. Rep. 2016; 6:20582 10.1038/srep20582 26838812 PMC4738326

[5] Sanchez DH, Lippold F, Redestig H, Hannah MA, Erban A, Krämer U, et al Integrative functional genomics of salt acclimatization in the model legume *Lotus japonicus*. Plant J. 2008; 53: 973–987. 10.1111/j.1365-313X.2007.03381.x 18047558

[6] Glenn EP, Brown JJ, Blumwald E. Salt tolerance and crop potential of halophytes. Crit. Rev. Plant Sci. 1999; 18: 227–255. 10.1080/07352689991309207

[7] Shabala S, Pottosin I. Regulation of potassium transport in plants under hostile conditions: implications for abiotic and biotic stress tolerance. Physiol. Plant. 2014; 151: 257–279. 10.1111/ppl.12165 24506225

[8] Assaha DV, Ueda A, Saneoka H, Al-Yahyai R, Yaish MW. The role of Na^+^ and K^+^ transporters in salt stress adaptation in glycophytes. Front. Physiol. 2017; 8:509 10.3389/fphys.2017.00509 28769821 PMC5513949

[9] Yamaguchi T, Blumwald E. Developing salt-tolerant crop plants: challenges and opportunities. Trends Plant Sci. 2005; 10: 615–620. 10.1016/j.tplants.2005.10.002 16280254

[10] Jamil A, Riaz S, Ashraf M, Foolad MR. Gene expression profiling of plants under salt stress. Crit. Rev. Plant Sci. 2011; 30: 435–458. 10.1080/07352689.2011.605739

[11] Roy SJ, Negrão S, Tester M. Salt resistant crop plants. Curr. Opin. Biotech. 2014; 26: 115–124. 10.1016/j.copbio.2013.12.004 24679267

[12] Munns R, James RA, Gilliham M, Flowers TJ, Colmer TD. Tissue tolerance: an essential but elusive trait for salt-tolerant crops. Funct. Plant Biol. 2016; 43: 1103–1113. 10.1071/FP16187 32480530

[13] Chinnusamy V, Jagendorf A, Zhu JK. 2005 Understanding and improving salt tolerance in plants. Crop Sci. 45, 437–448. 10.2135/cropsci2005.0437

[14] Verslues PE, Agarwal M, Katiyar-Agarwal S, Zhu J, Zhu JK. Methods and concepts in quantifying resistance to drought, salt and freezing, abiotic stresses that affect plant water status. Plant J. 2006; 45: 523–539. 10.1111/j.1365-313X.2005.02593.x 16441347

[15] Teng X, Cao W, Lan H, Tang H, Bao Y, Zhang H. *OsNHX2*, an Na^+^/H^+^ antiporter gene, can enhance salt tolerance in rice plants through more effective accumulation of toxic Na^+^ in leaf mesophyll and bundle sheath cells. Acta. Physiol. Plant 2017; 39:113 10.1007/s11738-017-2411-z

[16] Quan R, Wang J, Yang D, Zhang H, Zhang Z, Huang R. EIN3 and SOS2 synergistically modulate plant salt tolerance. Sci. Rep. 2017; 7:44637 10.1038/srep44637 28300216 PMC5353744

[17] Polle A, Chen S. On the salty side of life: molecular, physiological and anatomical adaptation and acclimation of trees to extreme habitats. Plant Cell Environ. 2015; 38: 1794–1816. 10.1111/pce.12440 25159181

[18] Flowers TJ. Improving crop salt tolerance. J. Exp. Bot. 2004; 55:307–319. 10.1093/jxb/erh003 14718494

[19] Zhao X, Wei P, Liu Z, Yu B, Shi H. Soybean Na^+^/H^+^ antiporter *GmsSOS1* enhances antioxidant enzyme activity and reduces Na^+^ accumulation in *Arabidopsis* and yeast cells under salt stress. Acta. Physiol. Plant 2017; 39:19 10.1007/s11738-016-2323-3

[20] Vij S, Tyagi AK. Emerging trends in the functional genomics of the abiotic stress response in crop plants. Plant Biotechnol. J. 2007; 5: 361–380. 10.1111/j.1467-7652.2007.00239.x 17430544

[21] Deyholos KM. Making the most of drought and salinity transcriptomics. Plant Cell Environ. 2010; 33: 648–654. 10.1111/j.1365-3040.2009.02092.x 20002333

[22] Haas BJ, Zody MC. Advancing RNA-seq analysis. Nat. Biotechnol. 2010; 28:421–423. 10.1038/nbt0510-421. 10.1038/nbt0510-421 20458303

[23] Smýkal P, Coyne CJ, Ambrose MJ, Maxted N, Schaefer H, Blair MW, et al Legume crops phylogeny and genetic diversity for science and breeding. Crit. Rev. Plant Sci. 2015; 34:43–104. 10.1080/07352689.2014.897904

[24] Chen C, Tao C, Peng H, Ding Y. Genetic analysis of salt stress responses in asparagus bean (*Vigna unguiculata* (L.) ssp. *sesquipedalis* Verdc.). J. Hered. 2007; 98: 655–665. 10.1093/jhered/esm084 17956901

[25] Win KT, Oo AZ. Genotypic difference in salinity tolerance during early vegetative growth of cowpea (*Vigna unguiculata* L.Walp.) from Myanmar. Biocatal. Agric. Biotechnol. 2015; 4: 449–455. 10.1016/j.bcab.2015.08.009

[26] Dong L, Ravelombola W, Weng Y, Qin J, Bhattarai G, Zia B, et al Seedling salt tolerance for above ground-related traits in cowpea (*Vigna unguiculata* (L.) Walp). Euphytica 2019; 215: 53 10.1007/s10681-019-2379-4

[27] Muñoz-Amatriaín M, Mirebrahim H, Xu P, Wanamaker SI, Luo M, Alhakami H, et al Genome resources for climate-resilient cowpea, an essential crop for food security. Plant J. 2017; 89: 1042–1054. 10.1111/tpj.13404 27775877

[28] Lonardi S, Muñoz-Amatriaín M, Liang Q, Shu S, Wanamaker S, Lo S, et al The genome of cowpea (*Vigna unguiculata* [L] Walp.). bioRxiv 2019 1 10.1101/518969 PMC6852540 31017340

[29] Li Y, Pan L, Wu H, Yu X, Liu Q, Guo R, et al Identification of salt tolerance of sixty asparagus bean (*Vigna unguiculata* (L.) ssp. *sesquipedalis* Verdc.) cultivars. Journal Plant Genet. Res. 2016; 17: 70–77. (in Chinese) 10.13430/j.cnki.jpgr.2016.01.011

[30] Bolger AM, Lohse M, Usadel B. Trimmomatic: a flexible trimmer for Illumina sequence data. Bioinformatics 2014; 30: 2114–2120. 10.1093/bioinformatics/btu170 24695404 PMC4103590

[31] Grabherr MG, Haas BJ, Yassour M, Levin JZ, Thompson DA, Amit I, et al Trinity: reconstructing a full-length transcriptome without a genome from RNA-Seq data. Nat. Biotechnol. 2011; 29: 644–652. 10.1038/nbt.1883 21572440 PMC3571712

[32] Kanehisa M, Furumichi M, Tanabe M, Sato Y, Morishima K. KEGG: new perspectives on genomes, pathways, diseases and drugs. Nucleic Acids Res. 2016; 45: D353–D361. 10.1093/nar/gkw1092 27899662 PMC5210567

[33] Kim D, Pertea G, Trapnell C, Pimentel H, Kelley R, Salzberg SL. TopHat2: accurate alignment of transcriptomes in the presence of insertions, deletions and gene fusions. Genome Biol. 2013; 14: R36 10.1186/gb-2013-14-4-r36 23618408 PMC4053844

[34] Trapnell C, Roberts A, Goff L, Pertea G, Kim D, Kelley DR, et al Differential gene and transcript expression analysis of RNA-seq experiments with TopHat and Cufflinks. Nat. Protoc. 2012; 7:562 10.1038/nprot.2012.016 22383036 PMC3334321

[35] Saeed AI, Sharov V, White J, Li J, Liang W, Bhagabati N, et al TM4: a free, open-source system for microarray data management and analysis. Biotechniques 2003; 34:374–378. 10.2144/03342mt01 12613259

[36] Du Z, Zhou X, Ling Y, Zhang Z, Su Z. agriGO: a GO analysis toolkit for the agricultural community. Nucleic Acids Res. 2010; 38: W64–W70. 10.1093/nar/gkq310 20435677 PMC2896167

[37] Dai X, Sinharoy S, Udvardi M, Zhao PX. PlantTFcat: An online plant transcription factor and transcriptional regulator categorization and analysis tool. BMC Bioinformatics 2013; 14:321 10.1186/1471-2105-14-321 24219505 PMC4225725

[38] Livak KJ, Schmittgen TD. Analysis of relative gene expression data using real-time quantitative PCR and the 2^−ΔΔCT^ method. Methods 2001; 25: 402–408. 10.1006/meth.2001.1262 11846609

[39] Thiel T, Michalek W, Varshney RK, Graner A. Exploiting EST databases for the development and characterization of gene-derived SSR-markers in barley (*Hordeum vulgare* L.). Theor. Appl. Genet. 2003; 106: 411–422. 10.1007/s00122-002-1031-0 12589540

[40] Pan L, Wang N, Wu Z, Guo R, Yu X, Zheng Y, et al A high density genetic map derived from RAD sequencing and its application in QTL analysis of yield-related traits in *Vigna unguiculata*. Front. Plant Sci. 2017; 8:1544 10.3389/fpls.2017.01544 28936219 PMC5594218

[41] Muchero W, Diop NN, Bhat PR, Fenton RD, Wanamaker S, Pottorff M, et al A consensus genetic map of cowpea [*Vigna unguiculata* (L) Walp.] and synteny based on EST-derived SNPs. Proc. Natl. Acad. Sci. USA 2009; 106: 18159–18164. 10.1073/pnas.0905886106 19826088 PMC2761239

[42] Kongjaimun A, Kaga A, Tomooka N, Somta P, Shimizu T, Shu Y, et al An SSR-based linkage map of yardlong bean (*Vigna unguiculata* (L.) Walp. subsp. *unguiculata sesquipedalis* group) and QTL analysis of pod length. Genome 2012; 55: 81–92. 10.1139/G11-078 22242703

[43] Xu P, Wu X, Muñoz-Amatriaín M, Wang B, Wu X, Hu Y, et al Genomic regions, cellular components and gene regulatory basis underlying pod length variations in cowpea (*V*. *unguiculata* L. Walp). Plant Biotechnol. J. 2017; 15: 547–557. 10.1111/pbi.12639 27658053 PMC5399003

[44] Schroeder JI, Allen GJ, Hugouvieux V, Kwak JM, Waner D. Guard cell signal transduction. Annu. Rev. Plant Bio. 2001; 52: 627–658. 10.1146/annurev.arplant.52.1.627 11337411

[45] Yang SS, Tu ZJ, Cheung F, Xu WW, Lamb JF, Jung HJG, et al Using RNA-Seq for gene identification, polymorphism detection and transcript profiling in two alfalfa genotypes with divergent cell wall composition in stems. BMC genomics 2011; 12: 199 10.1186/1471-2164-12-199 21504589 PMC3112146

[46] Demidchik V. Mechanisms of oxidative stress in plants: from classical chemistry to cell biology. Environ. Exp. Bot. 2015; 109: 212–228. 10.1016/j.envexpbot.2014.06.021

[47] Ouyang B, Yang T, Li H, Zhang L, Zhang Y, Zhang J, et al Identification of early salt stress response genes in tomato root by suppression subtractive hybridization and microarray analysis. J. Exp. Bot. 2007; 58: 507–520. 10.1093/jxb/erl258 17210988

[48] Hu L, Li H, Chen L, Lou Y, Amombo E, Fu J. RNA-seq for gene identification and transcript profiling in relation to root growth of bermudagrass (*Cynodon dactylon*) under salinity stress. BMC Genomics 2015; 16: 575 10.1186/s12864-015-1799-3 26238595 PMC4523028

[49] Schomburg FM, Bizzell CM, Lee DJ, Zeevaart JAD, Amasino RM. Overexpression of a novel class of gibberellin 2-oxidases decreases gibberellin levels and creates dwarf plants. Plant Cell 2003; 15:151–163. 10.1105/tpc.005975 12509528 PMC143488

[50] Magome H, Yamaguchi S, Hanada A, Kamiya Y, Oda K. The DDF1 transcriptional activator upregulates expression of a gibberellin-deactivating gene, *GA2ox7*, under high-salinity stress in *Arabidopsis*. Plant J. 2008; 56: 613–626. 10.1111/j.1365-313X.2008.03627.x 18643985

[51] Li H, Torres-Garcia J, Benhamed M, Schilderink S, Zhou W, Kulikova O, et al Plant-specific histone deacetylases HDT½ regulate GIBBERELLIN 2-OXIDASE 2 expression to control Arabidopsis root meristem cell number. Plant Cell 2017; 29: 2183–2196. 10.1105/tpc.17.00366 28855334 PMC5635991

[52] Lv S, Yu D, Sun Q, Jiang J. Activation of gibberellin 20-oxidase 2 undermines auxin-dependent root and root hair growth in NaCl-stressed *Arabidopsis* seedlings. Plant Growth Regul. 2018; 84: 225–236. 10.1007/s10725-017-0333-9

[53] Poitout A, Crabos A, Petřík I, Novák O, Krouk G, Lacombe B, et al Responses to systemic nitrogen signaling in *Arabidopsis* roots involve trans-zeatin in shoots. Plant Cell 2018; 30: 1243–1257. 10.1105/tpc.18.00011 29764985 PMC6048791

[54] Nacry P, Bouguyon E, Gojon A. Nitrogen acquisition by roots: physiological and developmental mechanisms ensuring plant adaptation to a fluctuating resource. Plant Soil. 2013; 370: 1–29. 10.1007/s11104-013-1645-9

[55] Buchner P, Hawkesford MJ. Complex phylogeny and gene expression patterns of members of the NITRATE TRANSPORTER 1/PEPTIDE TRANSPORTER family (NPF) in wheat. J. Exp. Bot. 2014; 65: 5697–5710. 10.1093/jxb/eru231 24913625 PMC4176842

[56] Bellegarde F, Herbert L, Séré D, Caillieux E, Boucherez J, Fizames C, et al Polycomb repressive complex 2 attenuates the very high expression of the *Arabidopsis* gene NRT2. 1. Sci. Rep. 2018; 8: 7905 10.1038/s41598-018-26349-w 29784958 PMC5962593

[57] Lai YS, Renna L, Yarema J, Ruberti C, He SY, Brandizzi F. Salicylic acid-independent role of NPR1 is required for protection from proteotoxic stress in the plant endoplasmic reticulum. Proc. Natl. Acad. Sci. USA 2018; 115: E5203–E5212. 10.1073/pnas.1802254115 29760094 PMC5984531

[58] Jayakannan M, Bose J, Babourina O, Rengel Z, Shabala S. Salicylic acid in plant salinity stress signaling and tolerance. Plant Growth Regul. 2015; 76:25–40. 10.1007/s10725-015-0028-z

[59] Smith TF, Gaitatzes C, Saxena K, Neer EJ. The WD repeat: a common architecture for diverse functions. Trends Biochem. Sci. 1999; 24: 181–185. 10.1016/S0968-0004(99)01384-5 10322433

[60] Kim YO, Kim JS, Kang H. Cold-inducible zinc finger-containing glycine-rich RNA-binding protein contributes to the enhancement of freezing tolerance in *Arabidopsis thaliana*. Plant J. 2005; 42: 890–900. 10.1111/j.1365-313X.2005.02420.x 15941401

[61] Kizis D, Lumbreras V. Role of *AP2/EREBP* transcription factors in gene regulation during abiotic stress. FEBS Lett. 2001; 498: 187–189. 10.1016/s0014-5793(01)02460-7 11412854

[62] Goyal E, Amit SK, Singh RS, Mahato AK, Chand S, Kanika K. Transcriptome profiling of the salt-stress response in *Triticum aestivum* cv. Kharchia Local. Sci. Rep. 2016; 6: 27752 10.1038/srep27752 27293111 PMC4904219

[63] Jiang Y, Deyholos MK. Functional characterization of *Arabidopsis* NaCl-inducible *WRKY25* and *WRKY33* transcription factors in abiotic stresses. Plant Mol. Biol. 2009; 69: 91–105. 10.1007/s11103-008-9408-3 18839316

[64] Yang O, Popova OV, Süthoff U, Lüking I, Dietz KJ, Golldack D. The *Arabidopsis* basic leucine zipper transcription factor *AtbZIP24* regulates complex transcriptional networks involved in abiotic stress resistance. Gene 2009; 436: 45–55. 10.1016/j.gene.2009.02.010 19248824

[65] Hou Y, Wu A, He Y, Li F, Wei C. Genome-wide characterization of the basic leucine zipper transcription factors in *Camellia sinensis*. Tree Genet. Genomes 2018; 14: 27 10.1007/s11295-018-1242-4

[66] Sun R, Ye R, Gao L, Zhang L, Wang R, Mao T, et al Characterization and ectopic expression of *CoWRI1*, an *AP2/EREBP* domain-containing transcription factor from coconut (*Cocos nucifera* L.) endosperm, changes the seeds oil content in transgenic *Arabidopsis thaliana* and rice (*Oryza sativa* L.). Front. Plant Sci. 2017; 8: 63 10.3389/fpls.2017.00063 28179911 PMC5263148

[67] Tian ZD, Zhang Y, Liu J Xie CH. Novel potato C2H2-type zinc finger protein gene, *StZFP1*, which responds to biotic and abiotic stress, plays a role in salt tolerance. Plant Biol. 2010; 12: 689–697. 10.1111/j.1438-8677.2009.00276.x 20701691

[68] Gourcilleau D, Lenne C, Armenise C, Moulia B, Julien JL, Bronner G, et al Phylogenetic study of plant Q-type C2H2 zinc finger proteins and expression analysis of poplar genes in response to osmotic, cold and mechanical stresses. DNA Res. 2011; 18:77–92. 10.1093/dnares/dsr001 21367962 PMC3077037

[69] Liu Q, Wang Z, Xu X, Zhang H, Li C. Genome-wide analysis of C2H2 Zinc-finger family transcription factors and their responses to abiotic stresses in poplar (*Populus trichocarpa*). PloS one 2015; 10: e0134753 10.1371/journal.pone.0134753 26237514 PMC4523194

[70] Sakamoto H, Maruyama K, Sakuma Y, Meshi T, Iwabuchi M, Shinozaki K, et al *Arabidopsis* Cys2/His2-type zinc-finger proteins function as transcription repressors under drought, cold, and high-salinity stress conditions. Plant Physiol. 2004; 136: 2734–2746. 10.1104/pp.104.046599 15333755 PMC523337

[71] Ciftci-Yilmaz S, Morsy MR, Song L, Coutu A, Krizek BA, Lewis MW, et al The ear-motif of the C2H2 zinc-finger protein ZAT7 plays a key role in the defense response of *Arabidopsis* to salinity stress. J. Biol. Chem. 2007; 282: 9260–9268. 10.1074/jbc.M611093200 17259181

[72] Park SJ, Kwak KJ, Oh TR, Kim YO, Kang H. Cold shock domain proteins affect seed germination and growth of *Arabidopsis thaliana* under abiotic stress conditions. Plant Cell Physiol. 2009; 50: 869–878. 10.1093/pcp/pcp037 19258348

[73] Li Y, Chu Z, Luo J, Zhou Y, Cai Y, Lu Y, et al The C2H2 zinc-finger protein Sl ZF 3 regulates AsA synthesis and salt tolerance by interacting with CSN 5B. Plant Biotechnol. J. 2018; 16: 1201–1213. 10.1111/pbi.12863 29193661 PMC5978872

[74] Xu T, Gu L, Choi MJ, Kim RJ, Suh MC, Kang H. Comparative functional analysis of wheat (*Triticum aestivum*) zinc finger-containing glycine-rich RNA-binding proteins in response to abiotic stresses. PloS one 2014; 9: e96877 10.1371/journal.pone.0096877 24800811 PMC4011930

[75] Yaxley JR, Ross JJ, Sherriff LJ, Reid JB. Gibberellin biosynthesis mutations and root development in pea. Plant Physiol. 2001; 125: 627–633. 10.1104/pp.125.2.627 11161020 PMC64864

[76] Li S, Fan C, Li Y, Zhang J, Sun J, Chen Y, et al Effects of drought and salt-stresses on gene expression in *Caragana korshinskii* seedlings revealed by RNA-seq. BMC Genomics 2016; 17: 200 10.1186/s12864-016-2562-0 26951633 PMC4782325

[77] Latz A, Mehlmer N, Zapf S, Mueller TD, Wurzinger B, Pfister B, et al Salt stress triggers phosphorylation of the *Arabidopsis* vacuolar K^+^ channel TPK1 by calcium-dependent protein kinases (CDPKs). Mol. Plant 2013; 6: 1274–1289. 10.1093/mp/sss158 23253603 PMC3971370

[78] Yang Y, Guo Y. Elucidating the molecular mechanisms mediating plant salt-stress responses. New Phytol. 2018; 217: 523–539. 10.1111/nph.14920 29205383

[79] Zhu J, Gong Z, Zhang C, Song CP, Damsz B, Inan G, et al OSM1/SYP61: a syntaxin protein in *Arabidopsis* controls abscisic acid-mediated and non-abscisic acid-mediated responses to abiotic stress. Plant Cell 2002; 14: 3009–3028. 10.1105/tpc.006981 12468724 PMC151199

[80] Jou Y, Chiang CP, Jauh GY, Yen HE. Functional characterization of ice plant SKD1, an AAA-type ATPase associated with the endoplasmic reticulum-golgi network, and its role in adaptation to salt stress. Plant Physiol. 2006; 141:135–146. 10.1104/pp.106.076786 16581876 PMC1459316

[81] Hamaji K, Nagira M, Yoshida K, Ohnishi M, Oda Y, Uemura T, et al Dynamic aspects of ion accumulation by vesicle traffic under salt stress in *Arabidopsis*. Plant Cell Physiol. 2009; 50: 2023–2033. 10.1093/pcp/pcp143 19880402

[82] Leshem Y, Golani Y, Kaye Y, Levine A. Reduced expression of the v-SNAREs AtVAMP71/AtVAMP7C gene family in *Arabidopsis* reduces drought tolerance by suppression of abscisic acid-dependent stomatal closure. J. Exp. Bot. 2010; 61:2615–2622. 10.1093/jxb/erq099 20423938 PMC2882261

[83] Sun X, Ji W, Ding X, Bai X, Cai H, Yang S, et al GsVAMP72, a novel *Glycine soja* R-SNARE protein, is involved in regulating plant salt tolerance and ABA sensitivity. Plant Cell Tiss. Org. 2013; 113:199–215. 10.1007/s11240-012-0260-4

[84] Tarte VN, Seok HY, Woo DH, Le DH, Tran HT, Baik JW, et al *Arabidopsis* Qc-SNARE gene AtSFT12 is involved in salt and osmotic stress responses and Na^+^ accumulation in vacuoles. Plant Cell Rep. 2015; 34: 1127–1138. 10.1007/s00299-015-1771-3 25689889

[85] Singh D, Yadav NS, Tiwari V, Agarwal PK, Jha B. A SNARE-like superfamily protein SbSLSP from the halophyte *Salicornia brachiata* confers salt and drought tolerance by maintaining membrane stability, K^+^/Na^+^ ratio, and antioxidant machinery. Front. Plant Sci. 2016; 7: 737 10.3389/fpls.2016.00737 27313584 PMC4889606

[86] Leshem Y, Melamed-Book N, Cagnac O, Ronen G, Nishri Y, Solomon M, et al Suppression of *Arabidopsis* vesicle-SNARE expression inhibited fusion of H_2_O_2_-containing vesicles with tonoplast and increased salt tolerance. P. Natl. Acad. SCI. USA 2006; 103:18008–18013. 10.1073/pnas.0604421103 17101982 PMC1693863

[87] Ravelombola W, Shi A, Weng Y, Mou B, Motes D, Clark J, et al Association analysis of salt tolerance in cowpea (*Vigna unguiculata* (L.) Walp) at germination and seedling stages. Theor. Appl. Genet. 2018; 131: 79–91. 10.1007/s00122-017-2987-0 28948303

